# Investigating the Effects of Tobacco Lignin on Polypropylene

**DOI:** 10.3390/polym14040706

**Published:** 2022-02-12

**Authors:** Irfan Tahir, John Rapinac, Abdulaziz Abutunis, Venkata Gireesh Menta

**Affiliations:** Mechanical & Industrial Engineering, University of Minnesota Duluth, Duluth, MN 55812, USA; tahir037@d.umn.edu (I.T.); rapin002@d.umn.edu (J.R.); abutu001@d.umn.edu (A.A.)

**Keywords:** lignin, polypropylene, tobacco lignin, blends, characterization, biopolymers

## Abstract

The utilization of eco-friendly materials, such as lignin, for higher value product applications became increasingly important as environmental concerns due to global warming increased. Melt blending is one of the easy ways to increase the usage of lignin in commercial applications. However, the degradation of the final product performance and increase in the production time and costs are of major concern. In the current work, the effects of blending lignin, extracted from tobacco plants, with polypropylene (PP) on the injection molding parameters, physical, thermal and mechanical properties are investigated. Blends of lignin (5, 15 and 30% by wt.) with PP were prepared using a Filabot single screw extruder. Results show that tensile strength decreases by 3.2%, 9.9% and 5.4% at 5 wt. %, 15 wt. %, and 30 wt. % of lignin addition, respectively. The tensile stiffness was almost unaffected by the addition of up to 15% lignin, but a 23% increase was observed at 30 wt. % loading. When compared to lignin processed via expensive processes, such as acetylation, tobacco lignin showed superior performance. The DSC results show unaffected crystallization and melting temperatures but a decrease in enthalpies and percentage of crystallinity. The SEM and optical micrographs of the coupon cross-sections show that the extrusion process has achieved a uniform distribution of lignin particles in the PP. Thermogravimetric analysis results show that tobacco lignin accelerates the onset decomposition temperature but does not influence the decomposition peak temperature. The increase in lignin content did not have a significant influence on the injection molding parameters, implying no additional processing costs for adding lignin to the PP. Overall, the performance of the tobacco lignin is comparable, if not better, than that of processed lignin reported in the literature.

## 1. Introduction

With an increase in the usage of plastics in applications ranging from household goods to 3D printed prototype parts, their impact on the environment is a growing concern. Using biopolymers, such as lignin, to substitute petroleum-based plastics is a widely accepted approach towards environmental sustainability. Derived from the Latin word lignum, meaning wood, lignin is an organic polymer found in the support tissues of plants, which is essential in the formation of plant cell walls [1]. After cellulose, it is the second most abundant renewable carbon source on Earth [2]. Annually, around 50 million tons of lignin are produced worldwide, as a by-product of mostly paper pulping industries, out of which only 2% is utilized commercially [3]. Recently, lignin has stirred interest among researchers, as it has shown to be one of the most promising candidates for blending with thermoplastic polymers [4]. Melt blending with petroleum-based plastics is one of the most convenient and inexpensive ways of using lignin. However, almost no work has been reported on lignin extracted from tobacco stalks, which generally goes into waste. As tobacco lignin can be obtained at a low cost and is fully biodegradable, replacing even part of petroleum-based plastics with lignin poses economic and environmental allure, especially if the physical properties and processing parameters of the blended products do not vary significantly. One such plastic is polypropylene (PP), which is used in various consumer products, such as household appliances, laboratory equipment, packaging materials and piping systems. It is estimated that the annual demand for PP will continue to grow from USD 76.00 billion in 2021 to USD 108.57 billion in 2028, at a compound annual growth rate (CAGR) of 5.2% [5]. 

Blending lignin with polymers has been studied for multiple decades now [6,7,8]. Kharade et al. reported the influence of lignin addition to PP on the mechanical properties [4]. The authors found that the tensile strength reduced with the increase in lignin content, while the impact properties remained constant and melt viscosity increased. Pucciariello et al. reported a significant reduction in strain but observed improvement in Young’s modulus [9]. Peng et al. studied the weathering and thermal properties of PP blended with wood flour, cellulose and lignin [10]. The authors found that the addition of lignin resulted in less of a reduction in flexural strength and modulus, compared to wood flour and cellulose. Alexy et al. studied the processing stability, mechanical properties and thermal properties of PP/lignin blends [11]. The authors found lignin to be a good processing stabilizer and an initiator for thermal degradation at higher concentrations.

Though lignin is a good candidate for biopolymer production, the high polarity and hydrophobicity of lignin makes it difficult to simply blend with other polymers and results in degrading the performance of the host polymer [6,7,8,9,10,11]. To improve the miscibility, procedures such as grafting acetyl or ethyl groups on hydroxyls, and usage of plasticizers is typical. Maldhure et al. reported significant improvements in mechanical properties by the addition of modified lignin to PP (up to 25 wt. %) compared to unmodified lignin [12]. Similar improvements in properties have been consistently reported by multiple authors, when secondary processes, such as esterification, alkalization, and co-grafting, are used [13,14,15]. However, these secondary processes are difficult to scale and increase the cost of lignin, making them unattractive to commercial adaptation. The objective of the current work is to investigate the usage of tobacco lignin as low-cost fillers in petroleum-based plastics, maximizing the percentage of renewable resources, while minimizing the processing modifications and loss of mechanical properties in the host polymer. Hence, lignin extracted from tobacco using a one-step process, without any secondary modifications or plasticizers, was identified and investigated.

Another major issue limiting the adaptability of lignin in commercial applications can be attributed to the wide variance in lignin quality. The recovery methods used are one of the key causes of the variability and poor performance of lignin. Commercially available lignin is typically obtained using procedures where cellulose and hemicellulose are the primary products and lignin is a byproduct. As a result, the lignin quality receives less attention. Processes aimed at generating high-quality lignin have the potential to alleviate a number of the difficulties raised. The lignin used in the present work is obtained using one such process, developed by Attis Innovations Inc., where the lignin is the primary product and the quality of lignin is the main focus. 

Tobacco has been one of the most important industries in at least three states (North Carolina, Georgia and Kentucky) in the USA and accounts for over 185,000 jobs. It is needless to list the harmful effects of tobacco products on human health. The current work aims to use the byproducts of harmful tobacco production to reduce the usage of petroleum-based polymers. Also, the effects of lignin, extracted from tobacco, on plastics have not been reported earlier. In the present study, lignin has been added to commercial PP at concentrations of 5 wt. %, 15 wt. % and 30 wt. %. Physical, mechanical and thermal properties of the blends were studied. Additionally, the effect of lignin addition on the processing parameters of PP was also investigated.

## 2. Materials and Methods

PP from M. Holland Inc. (Northbrook, IL, USA) in pellet form was used. The density of PP pellets was 0.90 g/cm^3^ and melt flow index (MFI) was 12 g/10 min. Lignin extracted from tobacco in the form of powder without any secondary modifications was obtained from Attis Innovations Inc., Milton, GA, USA. Attis Innovations Inc. claims to use a proprietary one-step butonal-organosolv processing technology to carefully extract and purify lignin, with consistent and high quality. No other additives were added to the mixture. Both materials were vacuum dried at 60 °C for 9 h to remove any moisture content. Materials were stored in a controlled environment.

PP pellets and tobacco lignin powder (5, 15, 30 wt. %) were weighed separately and placed in a container for thorough manual mixing for 5 min. The mixtures were then compounded at 180 °C in a Filabot EX2 single screw extruder (Filabot Inc., Barre, VT, USA). It has an extrusion rate of over 0.91 kg per hour. Extrusion parameters are as follows:

Nozzle diameter: 2.85 mm
Pellet size: 3.18 mm
L/D ratio: 12
Compression ratio: 2:5:1
Drive force: 9.6 Nm
Feed screw and drive: 35 RPM

The obtained filaments were then pelletized and re-extruded a second time to improve the dispersion of lignin particles in PP. The filaments were then pelletized into injection moldable pellets and stored in vacuum sealed containers.

**Figure 1:**
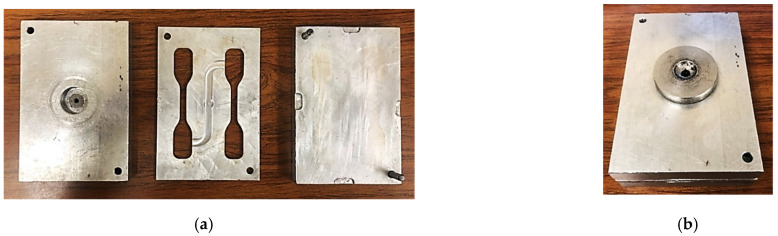
Three-piece injection mold designed for lignin blends: (**a**) showing the three separate pieces; (**b**) assembled mold with the sprue.

**Figure 2:**
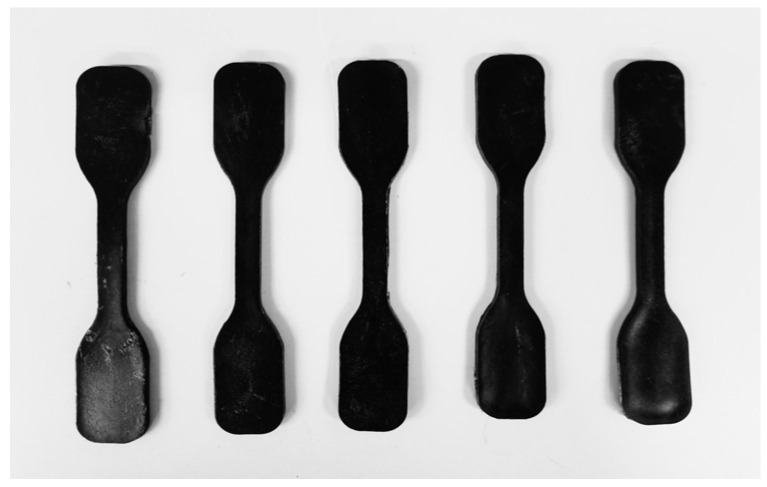
30 wt. % PP-lignin ASTM D638 Type IV tensile test coupons.

A Morgan Press G-125T injection molding machine (Morgan Industries Inc., Long Beach, CA, USA) was used for producing tensile test coupons following the ASTM D638 Type IV standard. A three-piece injection mold tool was designed and manufactured from Aluminum 6061 (see Figure 1) as lignin is typically known for its adhesion which can adversely affect the mold. Process parameters that resulted in parts without any defects such as short shots, warpage or flash were identified for neat PP and each lignin blend via trial and error process. Any required changes in processing parameters were recorded and the variations with change in lignin loadings were studied. Test coupons are shown in Figure 2. 

Thermal characterization of the blends was performed using a TA Instruments Discovery DSC 250 (TA Instruments, New Castle, DE, USA) in accordance with ASTM D3418-15. All the samples of about 5 mg were heated at a rate of 10 °C/min from 25 °C to 180 °C, held at constant temperature for 5 min, rapidly quenched to 25 °C, and then reheated to 180 °C (the second heating scan). Thermogravimetric analysis (TGA) was carried out for all PP/lignin blends to analyze thermal characteristics and long-term heat degradation of blend material using TA Instrument Discovery TGA 550 (TA Instruments, New Castle, DE, USA). The samples were subjected to a heating rate of 10 °C/min in the heating range of 40–800 °C. Once the mass loss plateau was established, the environment in the furnace was switched from inert to reactive oxygen environment. 

The density of the test coupons was measured using a Mettler Toledo precision balance following the ASTM D792 standard. Hardness tests were performed according to the ASTM D785-08 standard using a PTC Instrument shore D hardness tester. Tensile tests were performed on the test coupons using an Instron 5585H universal testing machine in accordance with ASTM D638 at a crosshead speed of 5 mm/min. Roller clamps were used to hold the coupons. Ultimate tensile strength (UTS) and Young’s modulus (E) were calculated for each test sample. At least five samples were tested at each blend percentile and the average values were reported with standard deviation for all the tests.

The cross-sections of the test specimens were analyzed using a Jeol JSM-6590LV Scanning Electron Microscope (SEM) from JEOL USA Inc., Peabody, MA, USA equipped with INCA X-act EDS detector for elemental analysis. Samples were bonded to aluminum stubs and their surfaces were observed in a low vacuum environment (30 Pa) using the accelerating voltage of 20.0 kV. Leica DVM6 optical microscope (Leica Microsystems Inc., Buffalo Grove, IL, USA) was also used to measure lignin particle size distribution to gain a better understanding of the blend morphology and its relation to the mechanical properties of the blend samples.

## 3. Results

### 3.1. Processing Stability of the Blends

Table 1 shows the injection molding parameters used for producing PP and PP-lignin blends. Other than increasing the injection pressure from 3500 psi to 4500 psi, no further changes were needed for producing the blend samples. Test coupons were fabricated easily, without any challenges, even at 30 wt. % of tobacco lignin loading and without any additives. Hence, adding lignin to PP does not require any major modifications to the current manufacturing process and does not incur additional major processing/utility costs.

### 3.2. Differential Scanning Calorimetry (DSC)

**Figure 3:**
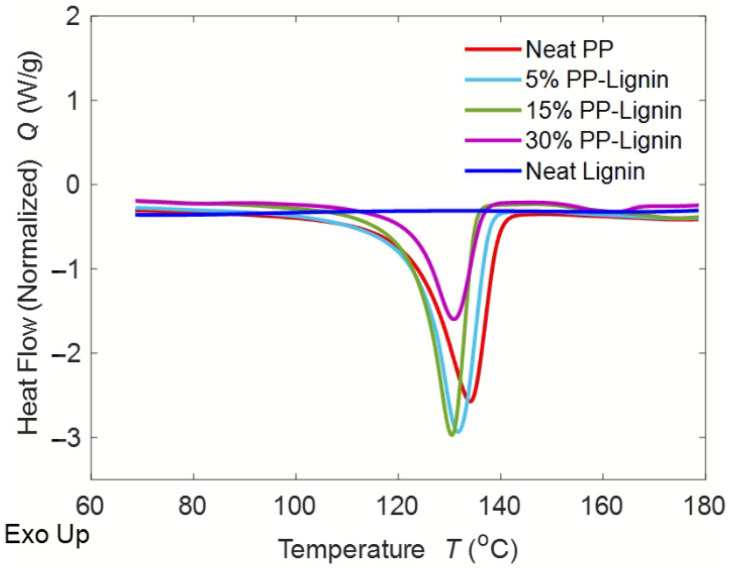
DSC Traces of PP/Lignin Blends.

**Figure 4:**
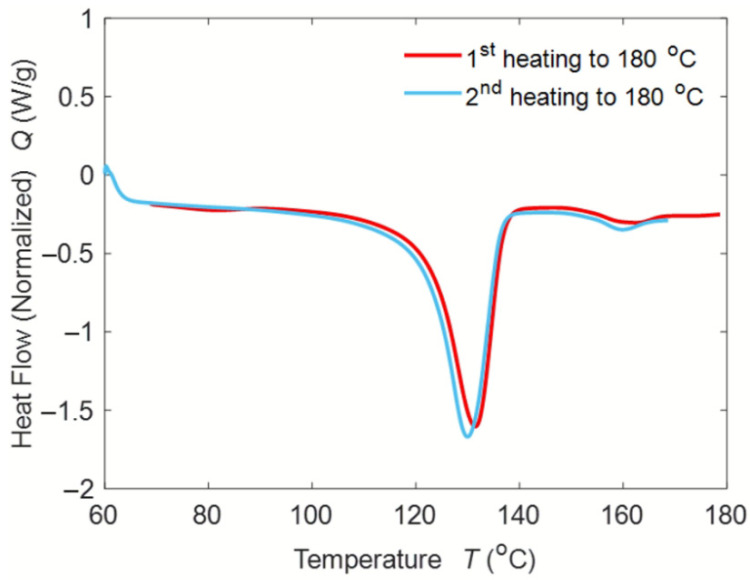
1st and 2nd heating curves of 30 wt. % PP-Lignin blends.

Figure 3 shows the DSC thermograms of PP, pure lignin and blends. Figure 4 shows the first and second heating curves of lignin blends at 30 wt. %. DSC is an acceptable method for measuring the glass transition temperature (T_g_) of lignin but it is often difficult to detect the T_g_ of lignin, due to the complexity of its chemical structure [16]. Typically, the T_g_ of lignin ranges from 90 °C to 180 °C. In this case, the T_g_ of lignin could not be identified. The crystallization temperature (T_c_), melt temperature (T_m_), exothermic enthalpy of crystallization (H_c_), and endothermic enthalpy of melting (H_m_) of the blends are reported in Table 2. As seen in the table, the peak melting and crystallization temperatures almost remained constant with the addition of lignin content. However, the exothermic and endothermic enthalpies gradually decreased with the increase in lignin content. The exothermic enthalpy dropped by 9%, 17%, and 50% at 5 wt. %, 15 wt. %, and 30 wt. % lignin addition, respectively. The endothermic enthalpy diminished by 11%, 22%, and 54%, at 5 wt. %, 15 wt. %, and 30 wt. % lignin addition, respectively. Typically, a decrease in enthalpies implies fewer bond formations and can significantly influence the behavior of the polymer. To further understand, the percentage of crystallinity in lignin blends was calculated by dividing the crystallization enthalpy by the heat of fusion from a perfect crystal of PP, as shown in the equation below:

$$\mathrm{Percent}\;\mathrm{crystallinity}\;\mathrm{equation}:\;X_{c}=\frac{∆H_{m}}{∆H{{}^{\circ}}_{m}}\times 100\%$$

where *X_c_* is the percentage of crystallinity, ∆*H_m_* is the measured heat of fusion from the crystallinity peak, and ∆*H*°*_m_* is the heat of fusion from a perfect crystal. The heat of fusion from a perfect crystal of PP is known to be 207 (J/g) [2]. The average percentage of crystallinity of PP and the lignin blends are shown in Table 2. The results show that the percentage of crystallinity gradually decreased with an increase in lignin content, which should typically translate into a significant reduction in the mechanical performance.

### 3.3. Thermogravimetric Analysis (TGA)

**Figure 5:**
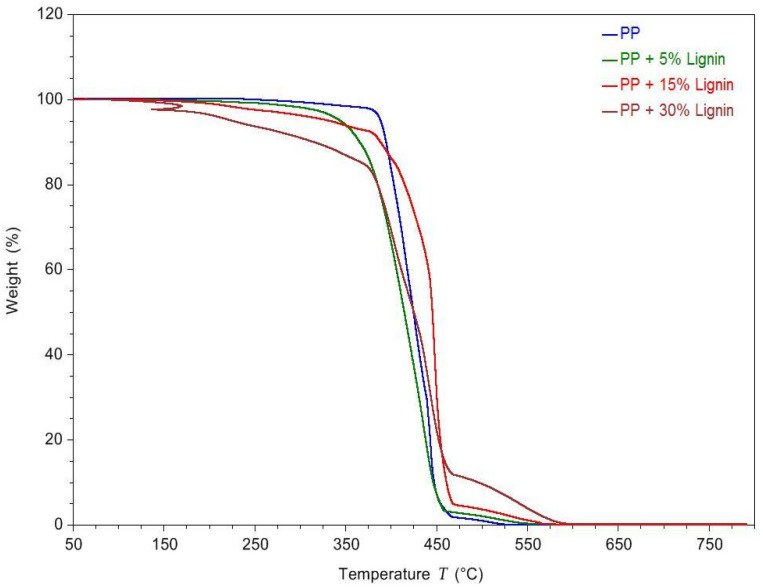
TGA curves for different blends of PP-lignin.

**Figure 6:**
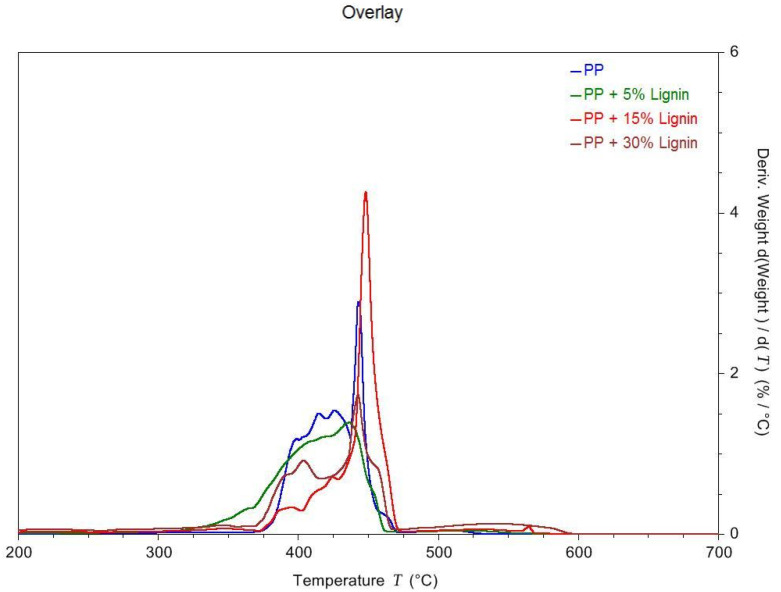
DTGA curves of different blends of PP-Lignin.

Figure 5 and Figure 6 show the TGA and DTGA curves of the lignin blends. A typical one-stage decomposition was observed for all the samples, except for the 30 wt. % PP-lignin blend [17]. Table 3 shows the decomposition onset temperature, end temperature, peak temperature and residual mass at 600 °C. The decomposition onset temperature is defined as the temperature at which 5 wt. % loss occurs. The temperature at which maximum weight loss is observed is the peak temperature of the derivative curve. From Figure 5, it can be seen that the degradation temperature of PP starts at 388.72 °C and continues to degrade until 451.58 °C. With the increase in the lignin content, the degradation temperature seems to drop to 344.12 °C at 5 wt. %, 331.27 °C at 15 wt. % and 224.61 °C at 30 wt. % lignin addition. A steady decrease in the decomposition onset temperature was observed with the increase in the lignin content in the PP. With only 5 wt. % addition of lignin, an 11% drop in the onset temperature was observed. Increasing the lignin content to 30 wt. % resulted in a 42% decrease in the onset temperature. Lignin typically has been considered to improve the thermal stability of the host polymers. However, tobacco lignin seems to have a destabilizing effect on the thermal degradation of blends, especially at higher concentrations [12]. 

### 3.4. Density

**Figure 7:**
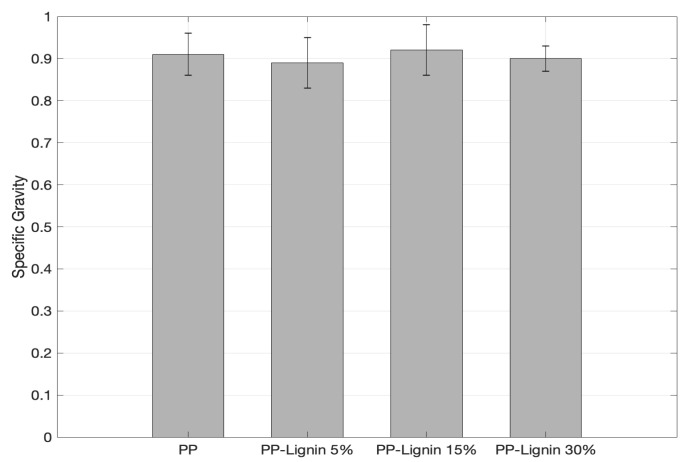
Density of Lignin Blends.

Figure 7 shows and compares the density of the blends. The density remains unchanged with the addition of lignin. With a significant decrease in the percentage of crystallinity from DSC, the density is expected to drop. However, the higher density of lignin appears to be compensating for the reduction in density due to lower crystallization. Similar behavior was observed with tensile performance, where only a slight reduction was observed, despite the significant decrease in the percentage of crystallinity. 

### 3.5. Shore D Hardness 

**Figure 8:**
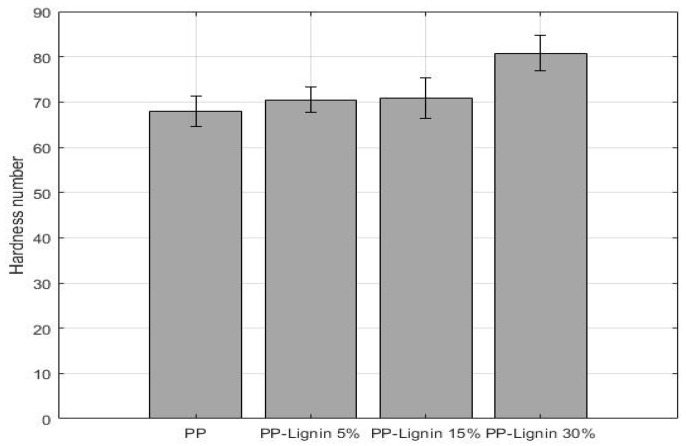
Shore D hardness values of lignin blends.

The Shore D hardness numbers for different blend specimens are presented in Figure 8. In all cases, the addition of lignin increased the hardness of composites. At 5 wt. % and 15 wt. % lignin loading, a slight increase of 3.7% and 4.1% was observed, respectively. However, the increase is within the error range and can statisticaly be considered as unchanged. However, at 30 wt. % lignin addition, an 18.82% increase in hardness value was recorded. These results are in close agreement with the tensile properties.

### 3.6. Tensile Properties

**Figure 9:**
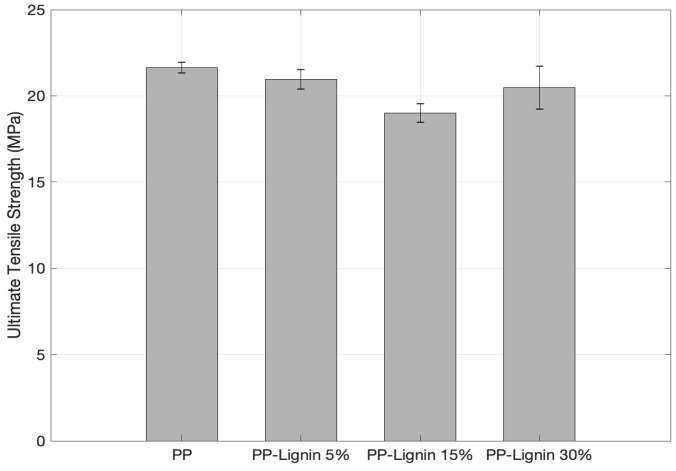
Ultimate tensile strength comparison (UTS).

The tensile properties of PP and lignin blends are shown in Table 4. Figure 9 compares the change in UTS as lignin wt. % is increased in PP-lignin blends. With an increase in 5 wt. %, 15 wt. %, and 30 wt. % of lignin, a drop of 3.2%, 9.9% and 5.4% in tensile strength was observed, respectively. Statistically, the values are within the standard deviation, and it can be stated that tensile strength seems to be unaffected by the addition of lignin. While a similar trend was observed with Young’s modulus, an exception in behavior was observed at 30% wt. lignin concentration, with a 20% increase in stiffness. The 30% wt. lignin blend samples also exhibited a brittle failure, unlike other samples. The variation in stiffness properties is very similar to ShoreD hardness values. 

Table 5 compares the tensile test results obtained in the current work with those from the literature. Kharade and Kale [4], who used dry lignin powder extracted from paper mill waste, found a drop of 59.3% when lignin concentration was increased to 30%. Toriz et al. [6], who used a purified form of kraft lignin, reported a drop of 37.4%. In the present work, a drop of only 5.4% was observed at 30 wt. % lignin loading to PP. Maldhure et al. [11], who used isolated kraft lignin from a paper mill, reported a 27.8% decrease in UTS for 15 wt. %, which is 15.6% higher than the one found in the present work. Overall, the tensile performance of tobacco lignin blends seems to be far superior compared to the ones reported in the literature. With the decrease in crystallinity, the tensile performance is supposed to deteriorate. Yet, a different behavior was observed. To investigate the driving forces behind this behavior, SEM and optical microscopy examinations were conducted. 

### 3.7. Scanning Electron Microscopy (SEM) and Optical Microscopy

**Figure 10:**
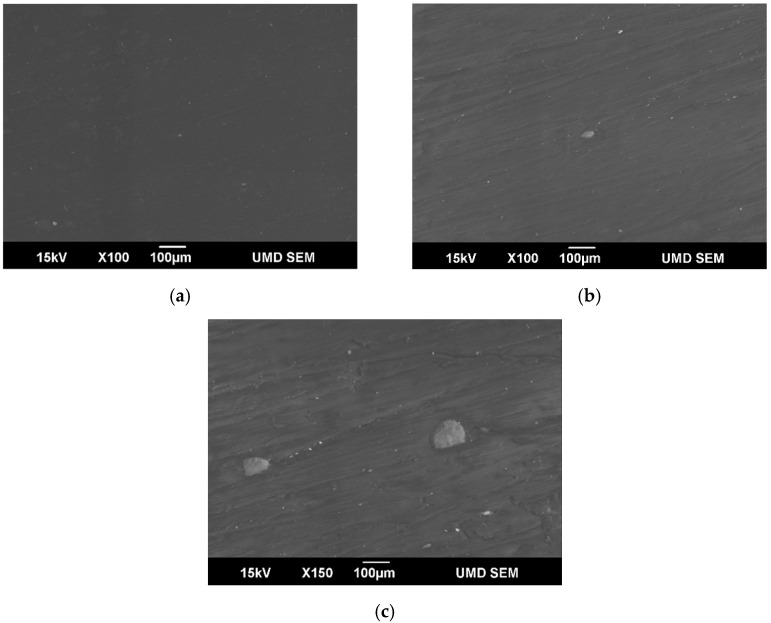
SEM Micrographs of (**a**) 5%, (**b**) 15% and (**c**) 30% PP-Lignin blends.

SEM micrographs of 5 wt. %, 15 wt. % and 30 wt. % blends are shown in Figure 10. At 5 wt. %, lignin seems to be well dispersed with particles barely being visible at 100× magnification. At 15 wt. %, a relatively larger sized lignin particles were observed. At 30 wt. %, more and larger aggregates can be seen. The strong polarity of lignin typically causes agglomeration, especially at high loading concentrations, and the mechanical characteristics decrease as the degree of agglomeration increases. Smaller lignin particles imply that lignin was successfully dispersed in PP [18,19]. 

**Figure 11:**
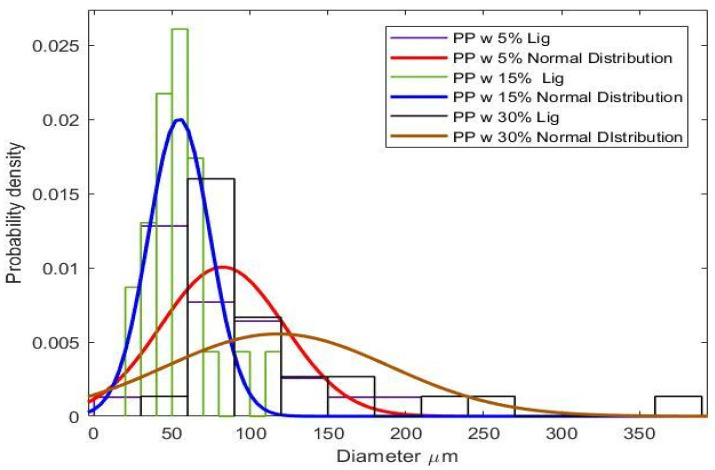
Probability density and histogram of tobacco lignin grain sizes.

Since dispersion plays an important role in the mechanical performance of the blends, optical microscopic images of fracture surfaces were taken, and Image J software was used to measure the size and density of lignin particles in the PP. A histogram with probability density curves is displayed in Figure 11. Particle sizes mainly ranged from 25–100 μm with a peak at 50 μm. Earlier works showed that the particle distribution of acetylated lignin was around 55 μm [11]. This implies a slightly better particle size distribution of tobacco lignin was achieved, without any secondary modifications to the lignin. The smaller lignin particle size and uniform dispersion can be attributed to an increase in mechanical performance of the blends, despite the decrease in crystallinity.

## 4. Conclusions

Physical and mechanical properties of PP/unmodified tobacco lignin (5%, 15% and 30% by wt.), prepared via melt mixing, were studied. The addition of lignin did not have an influence on the tensile strength, but with a 30 wt. % concentration of lignin, Young’s modulus increased by 23%. An increase in lignin did not influence the density and specific gravity of the blends. SEM images and optical micrographs of the coupon cross-sections showed that the extrusion process had achieved a uniform distribution of lignin particles. The addition of lignin did not require any modifications of injection molding parameters, except for a slight increase in the pressure, from 3500 psi to 4000 psi at 0 and 5 wt. %, implying that the addition of lignin will not increase any processing costs or delays. The DSC results showed that peak crystallization and melting temperatures did not change with the increase in lignin content, but the percentage of crystallinity decreased significantly. However, the unaffected mechanical properties can be attributed to better and uniform dispersion of lignin particles, along with a good adhesion between lignin and PP. The better mechanical performance also conveys that lignin particles might be acting as reinforcing material in the PP. However, further studies are warranted to understand the behavior better. TGA results showed that tobacco lignin acted as a thermal destabilizer at high temperatures. Comparing mechanical properties of unmodified tobacco lignin blends with those obtained from expensive acetylated lignin materials shows that the current material has superior properties. In conclusion, the addition of tobacco lignin up to 30 wt. % does not negatively affect the PP mechanical properties. Tobacco lignin shows great promise in improving some of the mechanical properties, without requiring any additional expensive processing steps.

## Figures and Tables

**Table 1:** Injection molding parameters.

Blend Composition	Barrel Temp (^°^F)	Nozzle Temp (^°^F)	Clamping Pressure (psi)	Plate Temp (^°^F)	Injection Pressure (psi)	Injection/ Dwell Time (s)
PP	420	450	7000	150	3500	5
5% Lignin	420	450	7000	150	4500	5
15% Lignin	420	450	7000	150	4500	5
30% Lignin	420	450	7000	150	4500	5

**Table 2:** DSC Test Results.

Blend Composition	T_c_ (°C)	Exothermic Enthalpy (J/g)	T_m_ (°C)	Endothermic Enthalpy (J/g)	Crystallinity (%)
Neat PP	115.67 ± 0.15	174.00 ± 0.69	132.07 ± 2.35	182.23 ± 3.52	84.06 ± 0.33
5% Lignin	115.50 ± 0.35	158.70 ± 0.57	133.69 ± 2.00	162.83 ± 4.04	76.67 ± 2.45
15% Lignin	115.37 ± 0.57	144.57 ± 0.73	131.73 ± 1.46	142.71 ± 5.50	69.84 ± 3.40
30% Lignin	114.83 ± 0.93	87.50 ± 0.74	132.10 ± 2.16	84.07 ± 1.88	42.27 ± 3.60

**Table 3:** Temperatures and Char Residue Characteristics of PP-Lignin Composties.

Blend Composition	T_onset_ (°C)	T_End_ (°C)	Decomposition Peak Temperature (°C)	Residual Mass at 600 °C (%)
Neat PP	388.72 ± 1.75	451.58 ± 0.97	444.01± 0.68	0.03
5% Lignin	344.12 ± 0.98	453.12 ± 1.45	440.35 ± 0.75	0.07
10% Lignin	331.27 ± 1.27	457.53 ± 2.14	448.78 ± 1.35	0.07
30% Lignin	224.61 ± 1.86	480.34 ± 1.78	441.80 ± 1.87	0.22

**Table 4:** Tensile properties of PP-lignin blend.

Blend Composition	Ultimate Tensile Strength (MPa)	Young’s Modulus (GPa)
Neat PP	21.65 ± 0.310	0.21 ± 0.02
5% Lignin	20.96 ± 0.570	0.21 ± 0.10
10% Lignin	19.50 ± 0.540	0.21 ± 0.03
30% Lignin	20.47 ± 1.25	0.24 ± 0.02

**Table 5:** Comparison of Ultimate Tensile Strength of PP-lignin with literature.

PP/Lignin	UTS % Decrease: Kharade and Kale [4]	UTS % Decrease: Toriz et al. ^1^ [6]	UTS % Decrease: Maldhure et al. [11]	UTS % Decrease: Present Work
95/5	19.5%	8.4%	16.2%	3.1%
85/15	28.1%	18.9%	27.8%	12.2%
70/30	59.3%	37.4%	-	5.4%

^1^ Calculated using interpolation as source work increased lignin in increments of 10.

## Data Availability

Not applicable.
